# The IES-R remains a core outcome measure for PTSD in critical illness survivorship research

**DOI:** 10.1186/s13054-019-2630-3

**Published:** 2019-11-19

**Authors:** Megan M. Hosey, O. Joseph Bienvenu, Victor D. Dinglas, Alison E. Turnbull, Ann M. Parker, Ramona O. Hopkins, Karin J. Neufeld, Dale M. Needham

**Affiliations:** 1 Department of Physical Medicine and Rehabilitation, Johns Hopkins School of Medicine, 600 North Wolfe Street, Phipps 179, Baltimore, MD 21287 USA; 2 Outcomes After Critical Illness and Surgery (OACIS) Group, Johns Hopkins School of Medicine, Baltimore, MD USA; 3 Department of Psychiatry and Behavioral Sciences, Johns Hopkins School of Medicine, Baltimore, MD USA; 4 Division of Pulmonary and Critical Care Medicine, Johns Hopkins University School of Medicine, Baltimore, MD USA; 5 Division of Pulmonary and Critical Care Medicine, Johns Hopkins School of Medicine, Baltimore, MD USA; 6 Department of Epidemiology, Johns Hopkins Bloomberg School of Public Health, Baltimore, MD USA; 7 Neuroscience Center and Psychology Department, Brigham Young University, Provo, UT USA; 8 Department of Medicine, Pulmonary and Critical Care Medicine, Intermountain Health Care, Murray, UT USA

To the Editor:

In response to Dr. Umberger’s comments [1] on the Impact of Event Scale-Revised (IES-R) [2] and the abbreviated 6-item IES (IES-6) [3], we offer guidance about assessing post-traumatic stress disorder (PTSD) symptoms as part of the existing National Institutes of Health-funded core outcome measurement set (COMS) for clinical research in acute respiratory failure (ARF) survivors [4].

An originator of the IES-R is no longer distributing the IES-R because PTSD diagnostic criteria have been revised in the Diagnostic and Statistical Manual of Mental Disorders, 5th Edition (DSM–5) [1]. Compared to DSM-IV, the DSM-5 separated the avoidance and numbing criteria and increased the number of associated symptoms from 17 to 20 [5]. However, this revision does not fundamentally change the phenotype of PTSD, and the IES-R/IES-6 continues to have utility in screening for PTSD symptoms [3, 5].

With respect to the above mentioned COMS, existing research has been highly heterogeneous in assessing PTSD, thus limiting advances in the field [4]. A 77-member international modified Delphi expert panel evaluated commonly used PTSD measures based on many criteria, including available psychometric evidence in ARF survivors, with a clear consensus recommendation to use the IES-R [4]. Diagnostic criteria for psychiatric disorders change frequently, without fundamental changes to the phenotype. Hence, without rigorous new research on PTSD screening in ARF survivors and another international consensus process, we do not endorse a unilateral change to the PTSD symptom measure recommended within the existing COMS (more information on the COMS is available at www.improveLTO.com).

In our communication with an originator of the IES-R regarding the above issues, his responses were “I consider it [IES-R] out of copyright …” and “There are enough [IES-R] copies floating around that you can more or less do whatever you want.” (Daniel S. Weiss, September 4, 2019) As evidence supporting the latter comment, we provide exemplar websites and publications that share the IES-R instrument (Table 1).

**Table 1:** Information on the Impact of Events Scale-Revised (IES-R) and IES-6 items and scoring

Instrument	Source	Access information
IES-R	Publication	Weiss DS, Marmar CR. The impact of event scale – revised. In Assessing Psychological Trauma and PTSD. 1997, 399–411. (page 408, Chapter 13*)
	Douglas Mental Health Institute and McGill University	http://www.info-trauma.org/flash/media-e/diagnosisToolkit.pdf (page 6 of pdf*)
	New York University Rory Meyers College of Nursing	https://consultgeri.org/try-this/general-assessment/issue-19.pdf (page 2 of pdf*)
	Publication	Beck JG, et al. The impact of event scale-revised: psychometric properties in a sample of motor vehicle survivors. *Journal of Anxiety Disorders*, 2008;22 (2) 187–198.
IES-6-item	Publication	Hosey et al. Screening for posttraumatic stress disorder in ARDS survivors: validation of the Impact of Event Scale-6 (IES-6). *Critical Care*, 2019; 23 (1), 1–7. https://ccforum.biomedcentral.com/articles/10.1186/s13054-019-2553-z

*Recommended approach for scoring IES-R based on existing IES-R psychometric publication in acute respiratory failure survivors (Bienvenu OJ, et al. Posttraumatic stress disorder in survivors of acute lung injury: evaluating the Impact of Event Scale-Revised. Chest, 2013; 144 (1), 24–31.) and Dr. Daniel S. Weiss (personal communication June 20, 2014):

• To obtain the three subscale scores (avoidance, hyperarousal, intrusion): calculate the mean of the subscale items

• To obtain a total score: calculate the mean of all non-missing items in the instrument

• IES-6-item is scored as mean value for these six items from IES-R

We hope that this information may provide helpful clarification in screening for PTSD symptoms in ARF survivors, a task that is essential for improving survivorship outcomes.

## Data Availability

Not applicable.
